# Shell structures in aluminum nanocontacts at elevated temperatures

**DOI:** 10.1186/1556-276X-7-115

**Published:** 2012-02-10

**Authors:** José Luis Costa-Krämer, Natalia León, Carlo Guerrero, Marisel Díaz

**Affiliations:** 1 IMM-Instituto de Microelectrónica de Madrid (CNM-CSIC), Isaac Newton 8, PTM, Tres Cantos, Madrid, E-28760, Spain; 2 Departamento de Mecánica, Universidad Simón Bolívar, Apartado Postal 89000, Caracas, 1080-A, Venezuela; 3 Centro de Física, Instituto Venezolano de Investigaciones Científicas (IVIC), Apartado Postal 20632, Caracas, 1020A, Venezuela

## Abstract

Aluminum nanocontact conductance histograms are studied experimentally from room temperature up to near the bulk melting point. The dominant stable configurations for this metal show a very early crossover from shell structures at low wire diameters to ionic subshell structures at larger diameters. At these larger radii, the favorable structures are temperature-independent and consistent with those expected for ionic subshell (faceted) formations in face-centered cubic geometries. When approaching the bulk melting temperature, these local stability structures become less pronounced as shown by the vanishing conductance histogram peak structure.

## Introduction

The study of shell structures in metallic nanowires has been a topic of increasing interest during the last few years [1-13]. These shell structures are a close analog to shell structures observed for metal clusters [14,15]. For alkali metals, it has been observed that the stability of the nanowires formed is influenced by electronic shell filling effects associated with atomic arrangements that produce the closing of electronic shells [1-3]. However, for Na and K at larger diameters, a crossover is found from which shell closings correspond to crystalline facet completion with an additional atomic layer [2,3]. These shell and subshell effects in nanowires manifest as stability peaks in the conductance histograms as the conductance depends on the wire's minimum cross section. In previous works on alkali metals [1-3], the conductance is measured at about one-third of the metal melting temperature as all the shell effect oscillations were observable at elevated temperatures. However, broad-peak precursors of the electronic shell effect are observed at 4.2 K [3]. In a previous work [4], clear evidence was presented of an atomic shell structure in gold nanocontacts formed at room temperature (far from the Au bulk melting point 1,340 K). These results confirmed conductance histograms as a powerful tool to study stable configurations of nanostructures.

In this work, conductance histograms for aluminum nanowires are presented at different temperatures, from room temperature [RT] up to near the melting point of the bulk. This explores the influence of temperature on the stable configurations seen by the different shell and subshell structures in these metal nanocontacts. This extends a previous study performed by a different technique at RT [16].

## Experiments

**Figure 1:**
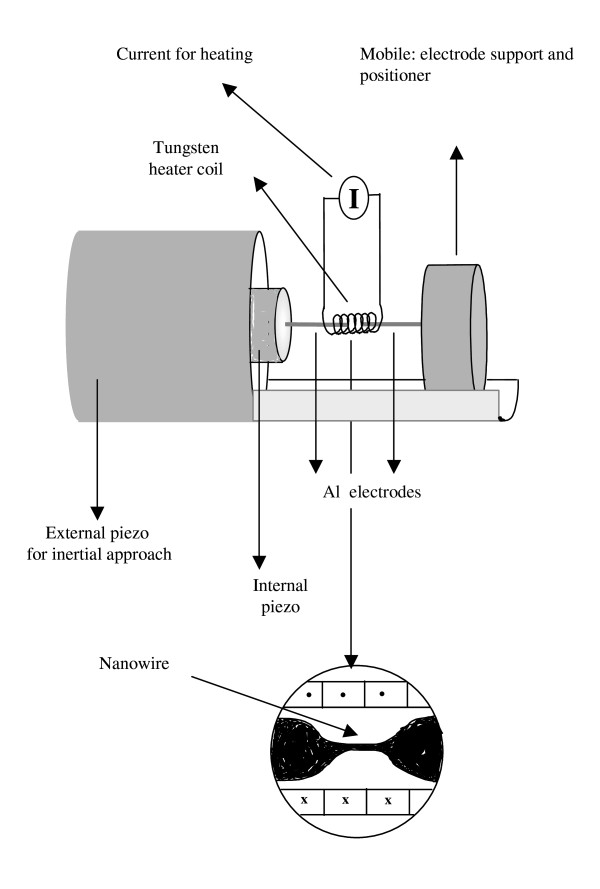
**Schematic experimental setup**. The Al nanowire is formed inside a tungsten heater coil, fed by a current source (I). The external piezo positions inertially one of the Al electrodes glued to the mobile, while the other Al electrode is glued to the internal piezo whose position is controlled by a voltage ramp.

Figure 1 shows the experimental setup. A custom-made scanning tunneling microscope is used to form and break contacts between two Al polycrystalline wires (99.95% purity). This is achieved by applying a voltage ramp, ± 100 V at 1 Hz, to an internal piezoelectric actuator which has glued one of the electrodes (Al wire). An external piezoelectric actuator holds, approaches, and separates controllably another Al wire glued to an inertial mobile. A tungsten heater coil tightly surrounds both electrodes. This heater is degasified before the experiments and used (1) to bake the electrodes prior to the experiments and (2) to maintain the electrodes at constant temperature during the nanowire elongation experiments. The setup is held in an ultrahigh vacuum chamber with a base pressure in the 10^-9 ^to 10^-10 ^Torr range. In addition, to ensure the maximum cleanliness of the Al contact area, every few minutes, a capacitor was charged with the electrodes separated and subsequently discharged across the electrodes by a controlled approach. This sublimates the Al contact area and creates two fresh surface contact areas. This discharge was visually observed from outside the chamber as a small spark in the contact area. Between cleaning procedures, series of 200 consecutive curves, lasting a few minutes, were acquired. This number was chosen because the dynamics of the contact formation/breakage at constant experimental conditions started degrading after 300 to 400 curves, probably signaling some local degradation/oxidation of the contact area. The current in the nanowire at a constant bias voltage is measured using a current-voltage converter with a 10^5 ^gain and an approximately 3-μs rise time. The current signal, triggered properly, is digitized and acquired with a TDS220 digital oscilloscope (Tektronix, Inc., Beaverton, OR, USA) and transferred to a personal computer through a GPIB where the conductance is calculated and displayed and the cumulative histogram of thousands of consecutive experiments is built.

## Results and discussion

**Figure 2:**
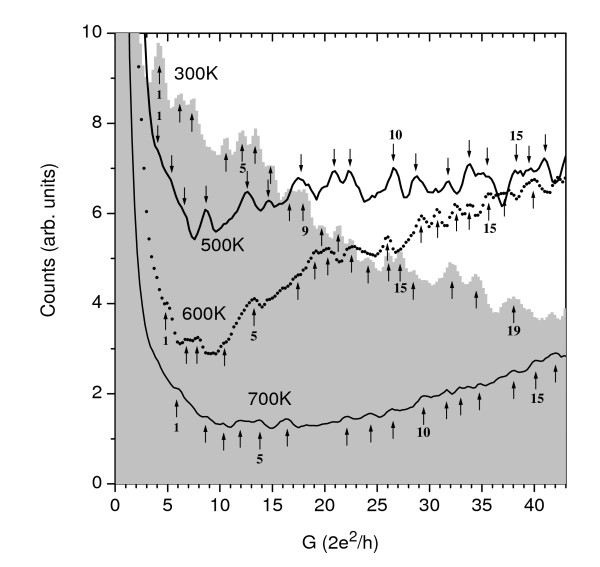
**Temperature dependence of conductance histograms up to 45 *G*_0 _for Al nanowires**. The histogram counts have been normalized to the total number of individual conductance traces (> 1,000 in all cases). The applied bias voltage between electrodes was 20 mV. Arrows indicate the set of identified main peaks for each temperature. The peaks are numbered as shown, and they are the index numbers, *m*, used in Figure 4.

**Figure 3:**
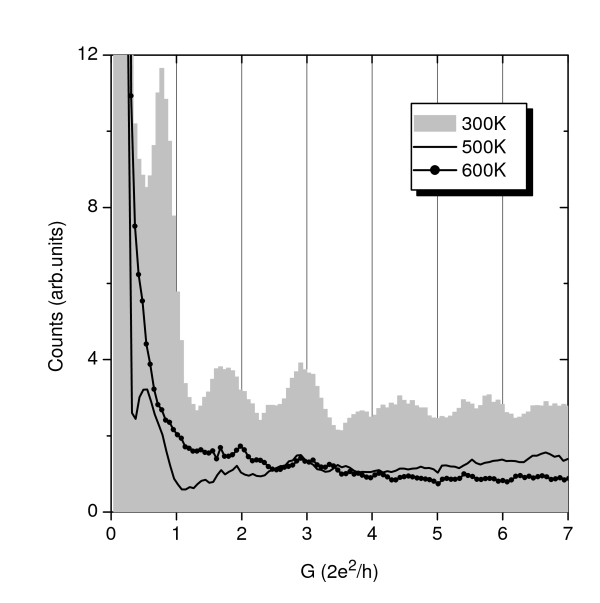
**Temperature dependence of conductance histograms up to 7 *G*_0 _for Al nanowires**. The histograms have been normalized to the total number of individual conductance traces (> 1,000 in all cases). The applied bias voltage between electrodes was 90 mV.

Figure 2 shows the Al nanowire conductance histograms recorded at a 20mV bias voltage for four different temperatures below the Al bulk melting point (930 K). The temperatures reported are estimations based on the oven temperature, measured with a pyrometer, and readjusted to match the observed melting of aluminum and indium wires of the same diameter placed at the electrode positions. Every histogram has been built with more than 1,000 individual conductance traces and normalized by their total number. These histograms allow to explore a region with *g *= *G*/*G*_0 _< 43 (where *G*_0 _= 2*e*^2^/h is the quantum of conductance). In Figure 3, we show the histograms obtained with a 90mV bias voltage. These histograms allow exploring the low conductance region (*g *< 7) with better resolution. By inspecting both figures, it is clear that the temperature has a dramatic effect on the histograms. At RT, it is clear that the presence of well-defined peaks close to quantum units (*g *at approximately 1, 2, 3). The peaks at *g *at approximately 2 and 3 have approximately the same intensity, as has been previously observed experimentally and supported by molecular dynamics simulations [17]. As the temperature increases, lower conductance peaks decrease in favor of broader and higher conductance structures. In fact, at 500 K, the peak at *g *at approximately 1 has one-third of the intensity of that at 300 K while the peak at *g *at approximately 3 is more intense than that at *g *at approximately 2 (see Figure 3). At 600 K, the peak at *g *at approximately 1 has already disappeared. It is due to increasing thermal instabilities. In addition, at high conductance values, the high conductance region of the histogram increases in comparison to the 300K case. This trend is similar to that found in alkali metals [1-3] and in molecular dynamics simulations of Al [18]. It is the result of the increased mobility of the atoms that allows the system to explore many atomic configurations in order to efficiently find a suitable local energy minimum.

**Figure 4:**
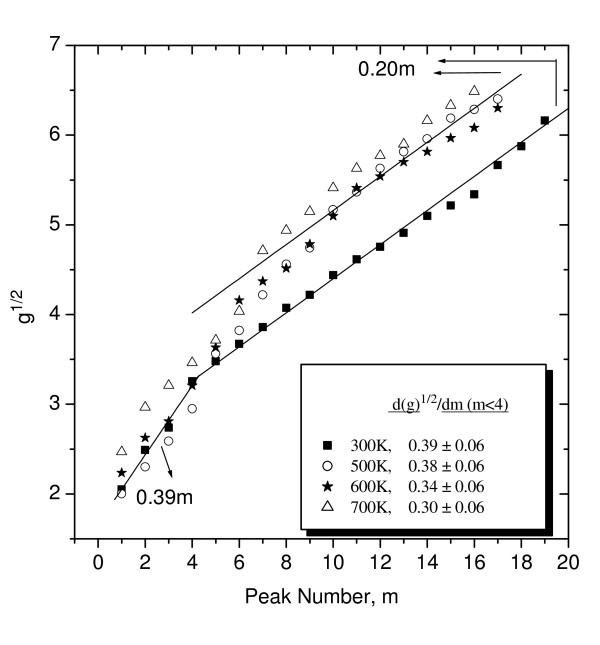
**Positions of the main peaks in Al conductance histograms**. Positions of the main peaks in Al conductance histograms (obtained from Figure 2) plotted against their sequential or peak index number *m*. Different straight lines and the value of their slopes are also displayed.

The presence of shell structures is revealed by a linear relationship between the wire radius, *R*, and the peak index number of the oscillations in the total energy of the wire that determine the stability of the structure [15,19]. The conductance of a nanowire is related to its radius through the Sharvin semiclassical expression [20], *g *= (*G*/*G*_0_) ~ (*k*_F_*R*/2)^2 ^to the first order. Then, in nanowires, these stable structures (i.e., cross sections) can be monitored by the conductance [1-4] as *g*^1/2 ^∝ *R*. A linear relationship between *g*^1/2 ^and the conductance peak number will indicate the existence of shells. Figure 4 shows *g*^1/2 ^versus the peak index number, *m*, that has been determined from the Al conductance histograms (Figure 2) as a function of the temperature. It should be taken into account that the first three peaks (see Figure 3) for *T *= 300 K were not considered as they are the result of the successive occupation of individual quantum modes leading to regular conductance quantization. The shift of the data to higher *g *values with the temperature is the result of the low-conductivity peaks' gradual disappearance; consequently, the indexation of the main peaks begins to shift to higher *g *values with the increase of the temperature (see Figure 2). Nevertheless, the presence of shell structures is revealed by a linear relationship between *g*^1/2 ^and the peak index number. Two markedly different behaviors are evident for all temperatures. For *m *> 4 (high conductance), the slope is 0.20 ± 0.02 in all cases. For *m *< 4 (low conductance), the slope is larger and seems to depend on the temperature; the obtained values are shown in Figure 4. However, the slope calculation in this range is somehow difficult since the peaks are less well defined, with a high degree of overlapping in some cases.

The results for the high-conductance region must be interpreted in terms of ionic subshells as for the larger structures, the dominance of crystal fields with respect to electronic effects must occur in all metals. The value of 0.20 ± 0.02 is well explained by optimal sections of a face-centered cubic [fcc] crystal, as previously has been proposed for the gold case at 300 K [4]. In fact, if the lattice structure of the wire is that of the bulk metal (fcc for Au and Al), at a large wire radius, a stable configuration is obtained each time a single facet of the wire cross section is completely covered with atoms, as suggested by Yanson et al. [2,3]. Two geometries for fcc wires produce similar results [4]. The first one has a hexagonal cross section with an axis oriented in the [011] direction and six equal area facets, four of which are hexagonally packed. The second energetically favored structure is octagonal, also with the axis along the [011] direction but with facets of exposed area fractions *β*_ijk_: *β*_111 _= 0.55, *β*_100 _= 0.25, and *β*_110 _= 0.20 accounting for both surface and edge energies. Using the conductance semiclassical expression, one can compute the conductance, *g*(*n*), as a function of the number of complete crystalline layers *n*. For the hexagonal section, the slope *d*(*g*^1/2^)/*dn *is 1.427 while 1.844 for the octagonal section. Such slopes are too high to explain the results, but if facets are filled individually, as previously suggested by Yanson et al. [2,3], the slopes decrease to 0.24 in the first case and to 0.23 in the second case. Both structures approach the measured value. Differences can be explained taking into account that experimentally, nanowires are generated from polycrystalline electrodes, so it is also expected that structures with other atomic arrangements or with an axis along directions different to [110] can contribute to the experimental value [5-10].

At low conductance values, the slopes seem to change with temperature (see data in the label in Figure 4), although as explained above, these data has to be treated carefully because it depends on the peak indexing procedure. However, it is quite clear that for aluminum, a very early (*m *≈ 4) crossover occurs to the ionic shell with fcc geometries for all temperatures; this is different to the gold case at 300 K where the crossover was observed at *m *≈ 8 [4]. In alkali metal nanocontacts, the low conductance slopes (between 0.54 for K and 0.62 for Li) have been associated with electronic shells by Yanson et al. [2,3]. For electronic shells, the results are explained in the semiclassical theory framework which shows that the periodicity is due to closed orbits of the delocalized valence electrons. Accordingly, the slope values are explained as the result of a combination of diametric, triangular, and square orbits (the first three shortest periodic orbits in a circular geometry which make only one revolution around the center). On the other hand, in earlier experiments on Al clusters, Lermé et al. [21] found a regular shell structure that they attributed to starlike orbits (the ones that make two turns around the center before closing). This orbit leads to a calculated slope of 0.33 to *g*^1/2^(*m*). However, Martin [15] has also found this regular shell structure in the mass spectrum of cold Al clusters and interpreted it in terms of ionic shells (filling of successive triangular facets of an octahedron). Our results in aluminum nanowires seem to show a slight decrease in the slope with temperature, from about 0.4 to 0.3 at moderate and elevated temperatures, respectively. However, due to the large error in the slope determination, which is partly due to the few points at low conductance, this slope might very well be constant and of about 0.35 for all temperatures. Therefore, our data cannot discern unambiguously the existence of temperature dependence of the *g*^1/2^(*m*) slope at low conductance.

In a previous work [17], the close correspondence between conductance and the number of atoms in the Al wire cross section was confirmed by comparing Al experimental conductance histograms at RT and the embedded atom in molecular dynamics simulations. Moreover, very recently, we have reported [22] both experimental results and molecular dynamics simulations of oscillations in Au and Al conductance histograms at RT. The intermediate and even the low-conductance slopes of Al experiments were well explained by the simulations. These results suggested that in Al nanocontacts, electronic shells were not evident at 300 K. Simulations performed by Gülseren et al. [6] suggest that thin aluminum wires should develop exotic, noncrystalline, stable atomic structures once their radii decrease below a critical size (5.3 Å in this case). Icosahedral packings or helical, spiral-structured wires may dominate these structures. For these wires, the surface energy dominates, resulting in a lower total energy compared to that of crystalline (fcc) wires and have been experimentally observed in gold [13] and platinum [23]. The experimental evidence presented in this work is not enough to establish if these types of structures are responsible of the behavior at low radii. The behavior is possibly due to a very early crossover between electronic and ionic shell filling effects. More experimental and theoretical studies on Al nanocontacts would be needed to understand the low conductance results.

## Conclusions

Evidence of subshell structures in the conductance histograms is presented for Al nanowires in the 300 to 700 K temperature range. The results for this metal imply a very early crossover from shell structures that dominate at a low wire radius to the ionic subshell structure at a larger radius. We have found that the structure at high radii (or conductance) values is independent on the temperature, and the results are consistent with those expected for ionic subshell (faceted) formation in fcc geometries. However, in approaching the bulk melting temperature, these local stability structures become less pronounced as shown by the vanishing conductance histogram peak structure. At low conductance values, a linear relationship between *g*^1/2 ^versus *m *is measured, although the data is not good enough to interpret the obtained values in terms of the electronic or ionic shells unambiguously.

## Competing interests

The authors declare that they have no competing interests.

## Authors' contributions

JLC-K fabricated the STM head, assembled the experimental UHV chamber, helped in carrying out the measurements, conceived the study, and participated in its design, data analysis, and coordination. NL and CG carried out most of the measurements and participated in the data discussion and analysis. MD assembled the experimental UHV chamber, carried out the measurements, conceived the study, and participated in its design, data analysis, and coordination. All authors read and approved the final manuscript.
